# Insulin Diminishes Superoxide Increase in Cytosol and Mitochondria of Cultured Cortical Neurons Treated with Toxic Glutamate

**DOI:** 10.3390/ijms232012593

**Published:** 2022-10-20

**Authors:** Vsevolod Pinelis, Irina Krasilnikova, Zanda Bakaeva, Alexander Surin, Dmitrii Boyarkin, Andrei Fisenko, Olga Krasilnikova, Igor Pomytkin

**Affiliations:** 1 Laboratory of Neurobiology, National Medical Research Center of Children’s Health, Russian Ministry of Health, Lomonosov Avenue 2, Bldg 1, 119991 Moscow, Russia; 2 Department of General Biology and Physiology, Kalmyk State University Named after B.B. Gorodovikov, St. Pushkin, 11, 358000 Elista, Russia; 3 Laboratory of Pathology of Ion Transport and Intracellular Signaling, Institute of General Pathology and Pathophysiology, Baltiyskaya St., 8, 125315 Moscow, Russia; 4 Department of Regenerative Medicine, National Medical Research Radiological Center, 4 Koroleva St., 249036 Obninsk, Russia; 5 Institute of Pharmacy, The First Sechenov Moscow State Medical University under Ministry of Health of the Russian Federation, St. Trubetskaya, 8, Bldg 2, 119991 Moscow, Russia

**Keywords:** insulin, glutamate (Glu) excitotoxicity, intracellular free Ca^2+^ concentration ([Ca^2+^]_i_), intracellular superoxide (O_2_^–•^), mitochondrial superoxide production, primary cortical neurons

## Abstract

Glutamate excitotoxicity is involved in the pathogenesis of many disorders, including stroke, traumatic brain injury, and Alzheimer’s disease, for which central insulin resistance is a comorbid condition. Neurotoxicity of glutamate (Glu) is primarily associated with hyperactivation of the ionotropic N-methyl-D-aspartate receptors (NMDARs), causing a sustained increase in intracellular free calcium concentration ([Ca^2+^]_i_) and synchronous mitochondrial depolarization and an increase in intracellular superoxide anion radical (O_2_^–•^) production. Recently, we found that insulin protects neurons against excitotoxicity by decreasing the delayed calcium deregulation (DCD). However, the role of insulin in O_2_^–•^ production in excitotoxicity still needs to be clarified. The present study aims to investigate insulin’s effects on glutamate-evoked O_2_^–•^ generation and DCD using the fluorescent indicators dihydroethidium, MitoSOX Red, and Fura-FF in cortical neurons. We found a linear correlation between [Ca^2+^]_i_ and [O_2_^–•^] in primary cultures of the rat neuron exposed to Glu, with insulin significantly reducing the production of intracellular and mitochondrial O_2_^–•^ in the primary cultures of the rat neuron. MK 801, an inhibitor of NMDAR-gated Ca^2+^ influx, completely abrogated the glutamate effects in both the presence and absence of insulin. In experiments in sister cultures, insulin diminished neuronal death and O_2_ consumption rate (OCR).

## 1. Introduction

L-Glutamate (Glu) serves as the main excitatory neurotransmitter in the central nervous system. Glu signaling is necessary for essential brain functions under normal conditions. However, excessive accumulation of extracellular Glu in a synaptic cleft and the space surrounding the neuronal soma can trigger a specific pathologic process known as excitotoxicity, which leads to the death of neurons. Glutamate excitotoxicity is involved in the pathogenesis of many disorders, including stroke, traumatic brain injury, and Alzheimer’s disease [1,2,3]. Excessive activation of ionotropic NMDA-type glutamate receptors (N-methyl-D-aspartate receptors, NMDAR), leading to a massive influx of Ca^2+^ and a biphasic increase in intracellular free Ca^2+^ concentration ([Ca^2+^]_i_), is considered to be central to the development of excitotoxicity [4,5,6,7]. Experiments with primary neuronal cultures have revealed that Glu causes a rapid moderate increase in [Ca^2+^]_i_ rise followed by a strong secondary [Ca^2+^]_i_ increase, called delayed calcium deregulation (DCD) [8,9]. DCD is supposed to be a point of no return in excitotoxicity. Any event that occurs downstream of the DCD onset is considered to influence the timing of cell death without altering its inevitability [8]. Long-lasting pathological changes in calcium homeostasis persisted in the surviving neurons after injury [10]. According to the data [4,6,9], DCD always occurs synchronously with a significant drop in the mitochondrial potential of the inner mitochondrial membrane (ΔΨm).

Studies of cultured neurons have shown that activation of NMDAR leads to an increase in intracellular superoxide anion radical (O_2_^–•^) and non-specified reactive oxygen species (ROS). Activation of NMDAR led to a rise in non-specified ROS in forebrain neurons [11], cortical neurons [12], striatal neurons [13], and spinal cord neurons [14]. In these studies, mitochondria have been suggested to be a major source of NMDAR-induced ROS increased O_2_^–•^ production upon activation of NMDAR was demonstrated in cultured neurons [15,16] and considered to cause excitotoxic cell death [17]. Several studies have provided evidence for mitochondria as a source of excitotoxic superoxide production [18,19]. Mitochondrial aconitase, the tricarboxylic acid cycle (TCA) enzyme, was identified as the main target of excitotoxic O_2_^–•^ in cortical cultures [17], which inactivation closely correlated with subsequent neuronal death [20]. However, it remains unclear how the increase evoked by NMDAR of [Ca^2+^]_i_ drive O_2_^–•^ formation in mitochondria.

There is evidence that nicotinamide adenine dinucleotide phosphate (NADPH) oxidase-2 (NOX2), rather than mitochondria, is the main source of NMDAR-induced superoxide production in cultured cortical and hippocampal neurons [21,22,23,24], as well as in the mouse hippocampus [23]. Interventions that prevent NOX2 activation have been shown to prevent excitotoxic cell death [21,22], indicating that NOX2 is a major source of excitotoxic O_2_^–•^. NMDAR-induced NOX2 activation requires not only an ionotropic Ca^2+^ influx but concomitant non-ionotropic NMDAR signaling through the association of phosphoinositide 3-kinase (PI3K) and the GluN2B subunit of NMDAR [25]. NOX2 is a transmembrane enzyme that functions as a transporter of electrons from cytoplasmic NADPH across membranes by the NOX2 protein complex to reduce molecular oxygen in the extracellular space or the lumen of intracellular organelles [26]. The membrane-bound NOX2 subunit gp91phox within dendrites was present near the surface membrane, vesicular organelles, and mitochondria [27]. Such a subcellular localization of the NOX2 complex in neurons indicates that NOX2 may produce superoxide both in the extracellular environment and in the intracellular compartments, including mitochondria. In general, there is a consensus on the importance of NMDAR activation and Ca^2+^ entry for the accumulation of toxic superoxide in neurons in excitotoxicity.

Recently, we have shown that insulin protects cultured cortical neurons from death and preserves neuron functions by decreasing the rise of glutamate-evoked [Ca^2+^]_i_ and preventing the onset of DCD [28]. However, the effects of insulin on glutamate-evoked O_2_^–•^ generation in neurons and the relationship between superoxide, NMDAR, and [Ca^2+^]_i_ have never been studied and are still to be clarified. The present study aims to investigate the effect of insulin on glutamate-induced O_2_^–•^ generation in cultures of rat cortical neurons, with an emphasis on the relationship between the dynamics of superoxide production and the increase in [Ca^2+^]_i_ in single neurons in response to the application of exogenous Glu [28].

## 2. Results

### 2.1. Changes in the Viability of Neurons under Glutamate Excitotoxicity and Insulin Treatment

**Figure 1:**
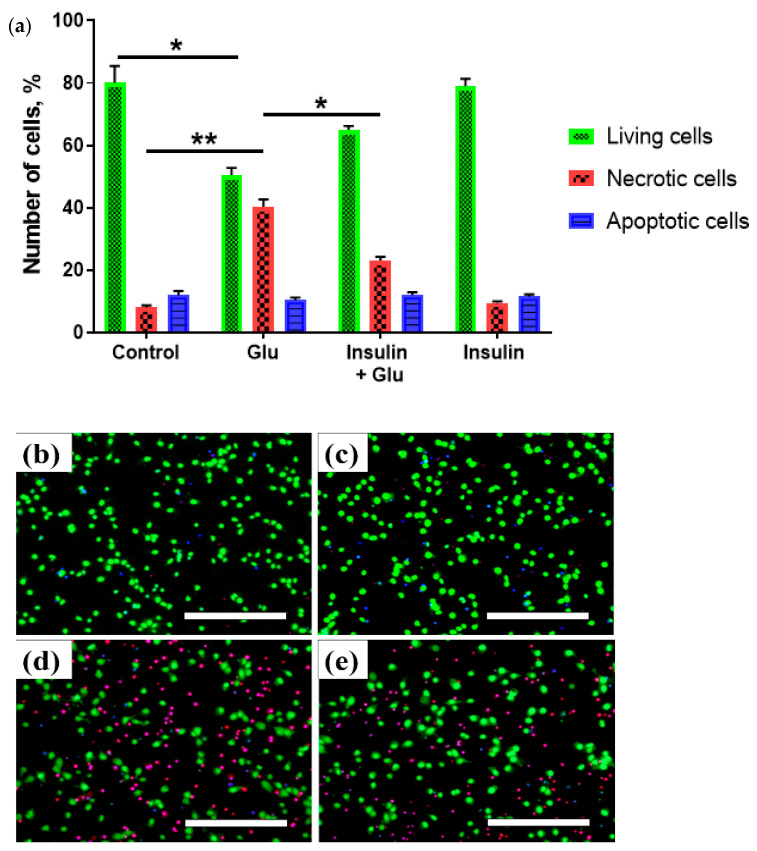
Insulin increases the proportion of living neurons and decreases the proportion of necrotic cells treated with the excitotoxic Glu dose. Proportion of cells (**a**) and fluorescent-microscopic photographs of the primary culture of rat cortical neurons one day after the excitotoxic action of glutamate (**b**–**e**). (**b**) control, (**c**) insulin, (**d**) glutamate, (**e**) glutamate with insulin. Cells were stained with vital fluorescent dyes (Syto-13, EthD-1, Hoechst-33342). Data are presented as M ± SEM. * *p* < 0.05; ** *p* < 0.001. The scale bar corresponds to 200 µm.

The use of fluorescent vital dyes revealed that Glu (100 µM) decreased the number of living cortical neurons by 29.7 ± 4.1% (*p* < 0.05) relative to the control (from 80.1 ± 8.3% to 50.4 ± 4.2%) (Figure 1). Ins alone (100 nm) did not affect cell survival. The pretreatment of cells with insulin 5 min before the application of Glu and then during the Glu treatment resulted in an increase in neuronal viability by 16.4 ± 3.3% (*p* < 0.001) compared to Glu. The increase in viability was associated with a decrease in the proportion of 1-positive cells, indicating a decrease in necrotic cells. 

### 2.2. Changes in Intracellular Calcium [Ca^2+^]_i_ and Superoxide

**Figure 2:**
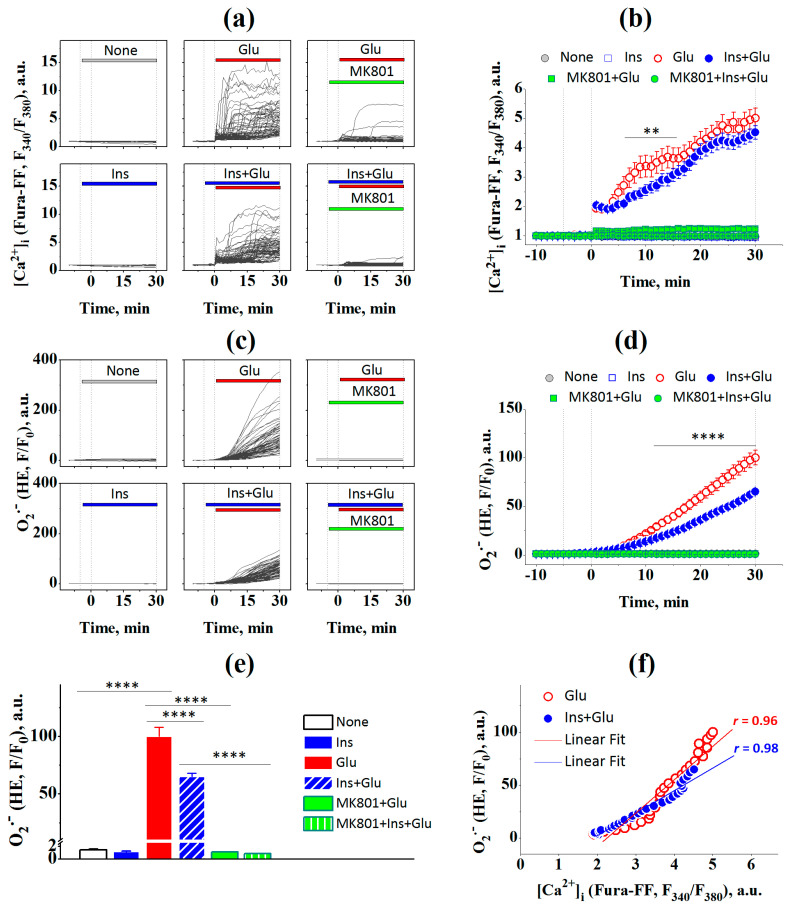
Insulin prevents the increase in intracellular superoxide ([O_2_^−•^]) and calcium ([Ca^2+^]_i_) in rat cortical neurons exposed to glutamate. (**a**) The dynamics of Fura-FF fluorescence, a measure of intracellular Ca^2+^, in single neurons (gray lines) treated with none (None; n = 103), 100 nM insulin (Ins; n = 94), 100 μM glutamate (Glu; n = 64), 100 nM insulin and 100 μM Glu (Ins + Glu; n = 72), MK-801 (10 μM) with 100 μM glutamate (MK801 + Glu; n = 100), and MK 801 with 100 nM insulin and 100 μM Glu (MK801 + Ins + Glu; n = 100). The data are obtained at every time point, at 60-second intervals, within 30 min of glutamate exposure. (**b**) The dynamics of Fura-FF fluorescence averaged over groups of neurons at every time point over a 30-min period. (**c**) The dynamics of HE fluorescence, a measure of intracellular [O_2_^−•^], in the same single neurons, gray lines. (**d**) The dynamics of HE fluorescence averaged over groups of neurons at every time point for a 30-min period. (**e**) Average levels of [O_2_^−•^] in neurons at the end of treatments. (**f**) Pearson’s correlations between the intracellular [Ca^2+^]_i_ means and [O_2_^−•^] of (**b**,**d**). Data of (**b**,**d**), and (**e**) are the mean ± SEM of the number of neurons, n. ** *p* < 0.01, **** *p* < 0.0001 (two-way ANOVA with repeated measures followed by Tukey’s post-test).

Primary cultured rat cortical neurons were coloaded with the low affinity fluorescent Ca^2+^ indicator Fura-FF and the fluorescent dye dihydroethidium (HE) to ensure simultaneous observation of changes in intracellular [Ca^2+^]_i_ and [O_2_^–•^], wherein intracellular [O_2_^–•^] includes both cytosol and mitochondrial superoxide. To investigate whether insulin can influence glutamate-evoked changes in intracellular Ca^2+^ and superoxide, neurons were treated with Glu (100 μM) in the presence or absence of insulin (100 nM), and the intracellular [O_2_^–•^] and [Ca^2+^]_i_ dynamics were monitored for 30 min (Figure 2). Two-way ANOVA with repeated measures revealed significant effects of time and insulin on the increase in both [Ca^2+^]_i_ (Figure 2b; F[40,21080] = 254.0, *p* < 0.0001 and F[5,527] = 116.6, *p* < 0.0001, respectively) and [O_2_^–•^] (Figure 2d; F[40,21080] = 519.1, *p* < 0.0001 and F[5,527] = 196.2, *p* < 0.0001, respectively). Tukey’s post-test showed that Glu-treated neurons had significantly higher levels of [Ca^2+^]_i_ (Figure 2b; from 1 to 30 min; *p* < 0.0001) and superoxide (Figure 2d; from 6 to 30 min; *p* < 0.001 to *p* < 0.0001) compared to those of intact neurons. At 30 min after the addition of Glu, the mean levels of [O_2_^−•^] were significantly higher (Figure 2e; *p* < 0.0001) compared to those in the intact control neurons. Insulin significantly decreased the Glu-evoked [Ca^2+^]_i_ (Figure 2b; from 6 to 15 min; *p* < 0.01 to *p* < 0.0001) and [O_2_^–•^] (Figure 2c; from 9 to 30 min; *p* < 0.05 to *p* < 0.0001). At 30 min, superoxide production in neurons treated with insulin and Glu was 35% lower (Figure 2e; *p* < 0.0001) than in neurons treated with glutamate alone. 

MK 801, a non-competitive NMDAR inhibitor, completely abolished Glu-induced increases in [Ca^2+^]_i_ and [O_2_^–•^], indicating a major role for NMDA receptors (Figure 2e; *p* < 0.0001). There were no statistically significant differences in [Ca^2+^]_i_ or [O_2_^–•^] between neurons treated with MK 801 and glutamate in the presence (*p* > 0.05) or absence of insulin (*p* > 0.05). These results suggest that NMDAR-gated Ca^2+^ influx is critically involved in the glutamate-evoked superoxide generation of superoxide in cortical neurons exposed to glutamate, regardless of the presence or absence of insulin. 

A significant Pearson linear correlation was found between the mean levels of [Ca^2+^]_i_ and intracellular superoxide levelsin neurons treated with glutamate in the absence of insulin (Figure 2f; r = 0.96, 95% confidence interval 0.91–0.97; slope 31.97 ± 1.84; F = 301.6, *p* < 0.0001) and in the presence of insulin (r = 0.98, 95% confidence interval 0.93–0.98; slope 21.17 ± 0.82; F = 666.1, *p* < 0.0001) within a 30 min period of exposure to Glu. 

### 2.3. Changes in [Ca^2+^]_i_ and the Mitochondrial Superoxide Production 

**Figure 3:**
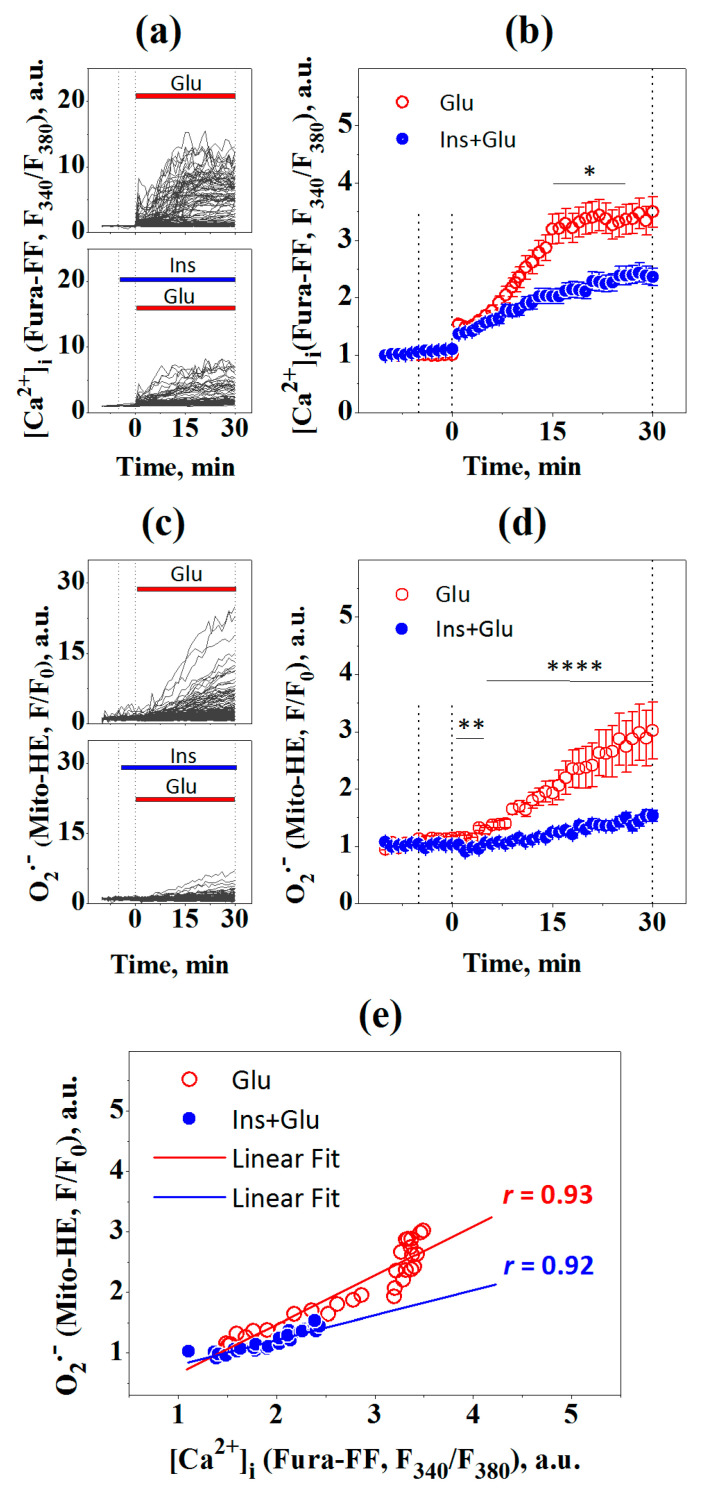
Insulin prevents the increase in mitochondrial superoxide ([O_2_^−•^]) in glutamate-exposed rat cortical neurons. (**a**) The dynamics of Fura-FF fluorescence, a measure of [Ca^2+^]_i_, in single neurons (gray lines) treated with 100 μM glutamate (Glu; number of neurons n = 149) or 100 nM insulin and 100 μM glutamate (Ins + Glu; n = 95). The data are obtained at every time point, at 60-s intervals, within 30 min of glutamate exposure. (**b**) The dynamics of Fura-FF fluorescence averaged over groups of neurons at every time point for a 30-min period. (**c**) Dynamic of Mito-HE fluorescence, a measure of mitochondrial superoxide, in the same single neurons, gray lines. (**d**) The dynamics of Mito-HE fluorescence averaged over groups of neurons at every time point over a 30-min period. (**e**) Pearson’s correlations between intracellular [Ca^2+^]_i_ means and mitochondrial superoxide of (**b**,**d**). Data for (**b**,**d**) are the mean ± SEM of the number of neurons, n. * *p* < 0.05, ** *p* < 0.01, **** *p* < 0.0001 (two-way ANOVA with repeated measures followed by Bonferroni’s post-test).

To investigate the effects of insulin on glutamate-induced production of mitochondrial superoxide, we used the mitochondrial-targeted fluorescent dye Mito-HE (a.k.a. MitoSOX Red). Rat cortical neurons were coloaded with Fura-FF and Mito-HE to simultaneously monitor changes in intracellular [Ca^2+^]_i_ and mitochondrial superoxide, respectively, and then treated with 100 μM glutamate in the presence or absence of 100 nM insulin (Figure 3). 

Two-way ANOVA with repeated measures revealed significant effects of time and insulin on glutamate-induced changes in both [Ca^2+^]_i_ (Figure 3b; F[40,9680] = 84.07, *p* < 0.0001 and F[1,242] = 7.63, *p* = 0.0062, respectively) and mitochondrial superoxide levels (Figure 3d; F[40,9680] = 44.41, p < 0.0001 and F[1,242] = 22.77, *p* < 0.0001, respectively). Bonferroni’s post-test shows that insulin significantly diminished glutamate-evoked rise in [Ca^2+^]_i_ (Figure 3b; from 15 to 24 min; *p* < 0.05) and mitochondrial superoxide levels (Figure 3d; from 1 to 30 min; *p* < 0.01 to *p* < 0.0001). At 30 min after treatment, the mean superoxide level in neurons treated with insulin and Glu was lower by 49% (*p* < 0.01) compared to neurons treated with Glu alone. There was a significant linear correlation between [Ca^2+^]_i_ and superoxide levels for Glu-treated neurons in the absence of insulin (Figure 3e; r = 0.93, 95% confidence interval 0.863–0.968; slope 0.80 ± 0.06; F = 188.96, *p* < 0.0001) and in the presence of insulin (r = 0.92, 95% confidence interval 0.834–0.961; slope 0.43 ± 0.04; F = 150.8, *p* < 0.0001). 

Thus, taken together, these results suggest that insulin prevents the glutamate-induced increase both in intracellular and mitochondrial superoxide production by diminishing the Glu-dependent rise in [Ca^2+^]_i_ in neurons. 

### 2.4. Effects of Glutamate and Insulin on Oxygen Consumption Rates in Neurons 

**Figure 4:**
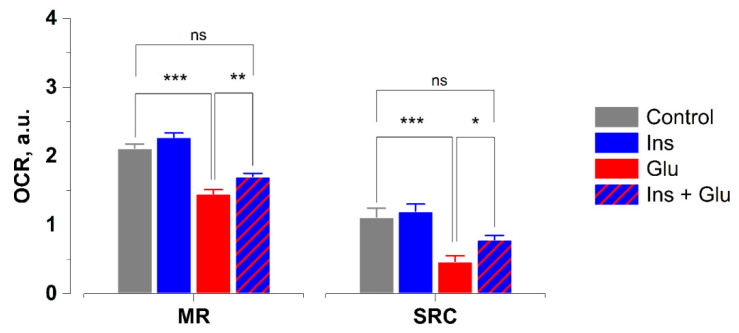
Insulin improves oxygen consumption rates (OCR) in rat cortical neurons exposed to glutamate. MR—maximal respiration. SRC—spare respiratory capacity. Data are the mean ± SEM of six to fourteen independent experiments. * *p* < 0.05, ** *p* < 0.01, *** *p* < 0.001 (Kruskal–Wallis followed by Dunn’s post-test). ns—non significant.

To elucidate the association between [O_2_^–•^] generation and oxygen metabolism during excitotoxicity of Glu, we measured oxygen consumption rates (OCR) in cortical neurons exposed to Glu (100 µM) in the presence or absence of insulin (100 nM). Kruskal–Wallis, followed by Dunn’s multiple comparison test, revealed that Glu exposure reduced maximal respiration by 31% (Figure 4; *p* < 0.001) and spare respiratory capacity by 58% (*p* < 0.001) compared to control. In agreement with the data shown in Figure 1c,d and Figure 2c,d, these results led us to suggest that the Glu-evoked rises in [Ca^2+^]_i_ and [O_2_^–•^] levels are concomitant with the glutamate-induced decrease in maximal respiration and SRC. Insulin significantly attenuated the inhibitory effects of Glu on maximal respiration (*p* < 0.01) and spare respiratory capacity (*p* < 0.05) compared to Glu alone. Since the latter relates to the amount of extra adenosine triphosphate (ATP) that can be produced by oxidative phosphorylation in response to increased energy demand, the protective effects of insulin against a glutamate-evoked increase in [Ca^2+^]_i_ and superoxide levels may be related to its preventive action against glutamate-induced impairment of mitochondrial bioenergetics, given that Ca^2+^, ATP, and superoxide exist in a network, with each able to control the others [28,29].

## 3. Discussion

Both insulin signaling disruption and Ca^2+^ dysregulation are closely related to memory loss during aging and increase the vulnerability to AD. Using the whole-cell patch clamp technique, Maimaiti et al. [30] showed that insulin decreases Ca^2+^ current through voltage-gated Ca^2+^ channels in hippocampal neurons, suggesting that insulin restores aging-related changes in Ca^2+^ regulatory pathways. Previously, we have reported that insulin protects cultured cortical neurons from death and preserves neuronal functions by reducing the Glu-evoked increase in [Ca^2+^]_i_ and preventing the onset of DCD [28]. In the present study, we demonstrated for the first time that insulin reduces the proportion of necrosis (DNA stained with EthD-1, Figure 1). The increase in neuronal survival is probably associated with a decrease in Glu-induced generation of both mitochondrial and non-mitochondrial [O_2_^–•^], the main sources of excitotoxic ROS in neurons (Figure 2 and Figure 3). 

Correlations close to linear were observed between mean [Ca^2+^]_i_ and [O_2_^–•^] when cultured cells were exposed to Glu alone or in the presence of insulin. Considering that insulin-suppressed Glu-induced secondary [Ca^2+^]_i_ rise (DCD), the inhibitory effect of insulin on [O_2_^–•^] accumulation appears to be related to its effect on [Ca^2+^]_i_ dynamics.

MK-801, an inhibitor of NMDAR-dependent Ca^2+^ influx, completely abrogated Glu-induced increase in [Ca^2+^]_i_ and [O_2_^–•^], regardless of the presence or absence of insulin, indicating a pivotal role for NMDA-type ionotropic glutamate receptors (Figure 2). Simultaneous measurements of changes in [Ca^2+^]_i_ and [O_2_^–•^] showed that the growth of [O_2_^–•^] occurs synchronously with the onset of DCD in each cell. Cessation of Ca^2+^ entry into the cell with the help of MK-801 abolished the formation of [O_2_^–•^] (Figure 2). Similar data were obtained in the absence of Ca^2+^ in the buffer (not shown). These results are consistent with previous data on the key role of Ca^2+^ influx through NMDAR in ROS accumulation during glutamate excitotoxicity.

Thus, the antioxidant effects of insulin on the glutamate-induced accumulation of unspecified ROS in differentiated human SH-SY5Y neuroblastoma cells have been previously shown [30]. However, the SH-SY5Y cell line does not appear to be a relevant model for mechanistic studies of oxidative stress in excitotoxicity since glutamate-induced ROS production in these cells occurs independently of NMDAR activation and Ca^2+^ influx [31]. The distinctive feature of the present investigation is that insulin effects on superoxide production were evaluated for the first time and were evaluated on the Glu excitotoxicity model in which excitotoxic superoxide production in vitro depends critically on the influx of Ca^2+^ driven by NMDAR in the same way as it occurs in vivo [23,24]. 

Using fluorescent probes, we found that insulin decreased the glutamate-evoked generation of both intracellular and mitochondrial superoxide. HE and Mito-HE fluorescent probes are believed to be appropriate for superoxide measurements in whole cells and mitochondria, respectively [31]. Due to its positive charge, Mito-HE preferentially accumulates ~1000-fold within the mitochondrial matrix [32]. However, in excitotoxicity, when mitochondrial depolarization occurs, Mito-HE partially leaks into the cytoplasm, and Mito-HE fluorescence can give overestimated mitochondrial superoxide values. A comparison of fluorescence intensities shows that HE fluorescence increased 47-fold, while Mito-HE fluorescence increased only 3-fold after 30 min of glutamate exposure (Figure 2 and Figure 3). This may indicate that NOX is the predominant producer of [O_2_^–•^]. However, a contribution of mitochondrial superoxide production to total [O_2_^–•^] production cannot be quantified from Mito-HE and HE fluorescence data, as these probes have different rates of oxidation with superoxide [33] and their fluorescence quantum yields are influenced by nucleic acid association [34]. Furthermore, despite its presumably smaller quantities, mitochondrial superoxide may play a critical role in excitotoxic cell death, as the inactivation of the tricarboxylic acid cycle enzyme aconitase by mitochondrial superoxide is closely correlated with cell death [17,20].

Based on our results from previous research [28] and this study, it is possible to draw a tentative conclusion about the mode of action of insulin on superoxide production in excitotoxicity. The main effect of insulin effect on superoxide production relates to preventing an excessive increase in [Ca^2+^]_i_. Before DCD development, NMDAR-gated Ca^2+^ influx is counterbalanced with mitochondrial Ca^2+^ uptake and its efflux produced by the plasma membrane Ca^2+^-ATPase and the Na+/Ca^2+^-exchanger (NCX). The development of DCD and profound mitochondrial depolarization cancel Ca^2+^ uptake by mitochondria and reverse F1Fo-ATP synthase to glycolytic ATP-consuming ATPase [4,5]. 

NCX [35] plays a key role in the excitotoxicity of Ca^2+^ because it has the highest transport capacity for Ca^2+^ [36,37]. NCX-mediated Ca^2+^ efflux is ATP-dependent since NCX exchanges one Ca^2+^ for three Na^+^, and the three Na+ are then pumped out by the Na^+^/K^+^ ATPase at the expense of one ATP. Prolonged exposure to glutamate leads to ATP depletion and a rise in [Ca^2+^]_i_ [28,29,38,39]. This rise becomes irreversible when a massive influx of Ca^2+^ is no longer counterbalanced by Ca^2+^ efflux due to NCX switching to reverse operating mode (NCXrev), which redirects the flow of Ca^2+^ into the cell [37,40]. Therefore, maintaining the maintenance of ATP production is critical for preventing a rise in [Ca^2+^]_i_ in excitotoxicity. 

Recently, we have shown that glutamate-induced ATP depletion occurs concomitantly with comparable decreases in OCR, maximal respiration, and spare respiratory capacity (SRC) [28]. Insulin decreased glutamate-evoked ATP depletion and SRC. SRC is potentially related to the amount of extra ATP that can be produced by oxidative phosphorylation in case of increased energy demand [33,41]. Therefore, the maintenance of mitochondrial bioenergetics and, primarily, SRC seems to be central to insulin effects during glutamate excitotoxicity [42]. It has long been known that the tricarboxylic acid cycle is the intracellular site of insulin action [41,43]. Insulin stimulates succinate oxidation in mitochondrial complex II, which has been identified as the main source of SRC [41]. In this study, we found that Glu induces an increase in [Ca^2+^]_i_ and superoxide levels concomitantly with the decrease in SRC (Figure 4). Insulin mitigates the glutamate effect on SRC and reduces the glutamate-evoked increase in [Ca^2+^]_i_ and superoxide levels. In our previous report, we demonstrated that insulin improved SRC in glutamate-treated neurons, thereby reducing glutamate-evoked ATP depletion [28], where SRC refers to the amount of extra ATP that can be produced via oxidative phosphorylation in the case of increased energy demand. Therefore, the maintenance of mitochondrial bioenergetics and—primarily—SRC seems to be central to insulin’s inhibitory effects on superoxide production during glutamate excitotoxicity. Insulin effects on superoxide generation during glutamate excitotoxicity seem to be related to maintaining oxidative phosphorylation in mitochondria. Insulin counteracts glutamate-evoked ATP depletion, thus preventing the glutamate-evoked rise in [Ca^2+^]_i_ and the subsequent increase in superoxide generation. 

**Figure 5:**
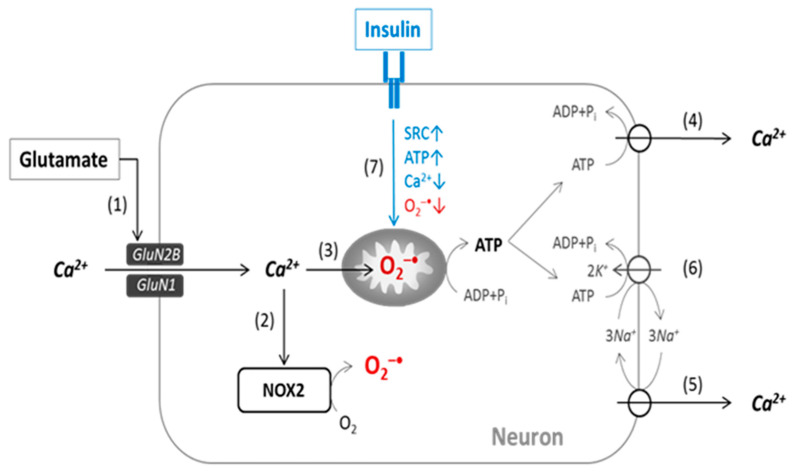
Scheme for a functional relationship between NMDAR and the insulin receptor (IR) during superoxide generation in excitotoxicity. After glutamate-evoked NMDAR activation (1), Ca^2+^ entry leads to an increase in superoxide [O_2_^−•^] generation at NOX2 (2) and a rise in superoxide in the mitochondrial matrix (3). The NMDAR-gated Ca^2+^ influx is counterbalanced by ATP-dependent Ca^2+^ efflux mediated by plasma membrane Ca^2+^-ATPase (4) and the Na^+^/Ca^2+^-exchanger (NCX), (5) coupled with Na^+^/K^+^-ATPase (6). Following Ca^2+^ entry into the mitochondrial matrix, ATP depletion occurs, thus affecting the ATP-dependent efflux of Ca^2+^ and leading to a rise in [Ca^2+^]_i_. Insulin significantly increases spare respiratory capacity SRC (7), thus maintaining ATP production and improving ATP-dependent Ca^2+^ efflux. This prevents [Ca^2+^]_i_ from rising, which in turn reduces superoxide generation in NOX2 and in mitochondria.

Figure 5 presents a scheme illustrating the functional relationship between NMDAR and insulin receptor signaling during glutamate-evoked superoxide generation. The results received allowed us to confirm that insulin’s neuroprotective effect on glutamate excitotoxicity is a consequence of its action in a decrease in [Ca^2+^]_i_, an increase in [ATP]_i_, and a decrease in mitochondrial and cytosolic superoxide formation. 

Short-term insulin exposure in our experiments appears to be relevant for in vivo conditions of intranasal insulin therapy. Intranasally managed insulin has been shown to reach a peak value in the rat brain 15 min after administration [44]. The rapid inhibitory effect of insulin on the onset of oxidative stress observed in our study seems to explain, at least in part, the neuroprotective action of intranasal insulin in the treatment of Alzheimer’s disease [45] and experimental traumatic brain injury [46]. In both cases, oxidative stress caused by glutamate excitotoxicity is a concomitant condition or symptom. 

We found that short-term exposure to insulin prevents glutamate-evoked superoxide generation in cultures of rat cortical neurons by diminishing the glutamate-induced rise in [Ca^2+^]_i_ and (ii) decreasing the superoxide yield rate by the same increment in [Ca^2+^]_i._ The effects of insulin on glutamate excitotoxicity are associated with improved spare respiratory capacity and the ability to produce additional ATP due to oxidative phosphorylation. Given that generation of [O_2_^–•^] generation is considered to be causal for neuronal death, the prevention of the onset of oxidative stress with insulin appears to at least partially explain its neuroprotective action in glutamate excitotoxicity. 

An unexpected finding of our study is that the observed value of increased superoxide level, at the same increment as the increase of [Ca^2+^]_i_, was on average 32% and 46% lower in insulin-treated neurons, estimated using HE and Mito-HE. This suggests that the increase of [Ca^2+^]_i_, while necessary, is not the only factor affecting the magnitude of superoxide production. Given a recent finding that NMDAR-induced superoxide production requires non-ionotropic NMDAR signaling via PI3K, the inhibitory effect that insulin has on superoxide generation may perhaps be related to interference between insulin receptor [47] and non-ionotropic NMDAR signaling [25], as PI3K is a common signaling molecule in both pathways. However, the explanation for this effect must be the subject of future research.

## 4. Materials and Methods

### 4.1. Primary Culture of Rat Cortical Neurons

All reagents were obtained from Invitrogen (Thermo Fisher Scientific, Waltham, MA, USA) or Sigma-Aldrich (Merck, St. Louis, MO, USA). Cell culture supplies were obtained from Invitrogen (Thermo Fisher Scientific, Waltham, MA, USA).

Experiments with animals were carried out according to the ethical principles and regulatory documents recommended by the European Convention for the Protection of Vertebrate Animals used for Experimental and other Scientific Purposes, as well as by the “Good Laboratory Practice Rules”, approved by order of the Russian Federation Ministry Health No. 199 n from 4 January 2016. Primary cultures were prepared from the cortex of one- or two-day-old Wistar rats as previously described [28]. The rats were anesthetized and decapitated, the skull was opened, the brain was withdrawn, and then the cortex was isolated by removing the membranes. The extracted tissues were washed in Hanks’ solution without Ca^2+^ and Mg^2+^ (Gibco; Thermo Fisher Scientific, Inc., Waltham, MA, USA) with 0.04% NaHCO_3_ (HBSS), ground, and placed for 15 min in a 0.05% papain solution in medium (10 mg L-cysteine HCl, 10 mg BSA, 250 mg glucose with 0.02% EDTA and 5 µL DNAse). After incubation in papain, the altered mixture was washed twice with standard Hank’s solution (Gibco; Thermo Fisher Scientific, Inc., Waltham, MA, USA) with phenol red and then with Minimal Essential Medium culture medium (MEM; Gibco; Thermo Fisher Scientific, Inc., Waltham, MA, USA). The cells were then dispersed in a fresh MEM medium until a homogeneous suspension was obtained, which was pelleted twice in a centrifuge at 200× *g*. The precipitated cells were resuspended in an appropriate volume of Neurobasal Medium (NBM; Gibco; Thermo Fisher Scientific, Inc., Waltham, MA, USA) supplemented with Supplement B-27 (Gibco; Thermo Fisher Scientific, Inc., Waltham, MA, USA), GlutaMax (Gibco; Thermo Fisher Scientific, Inc., Waltham, MA, USA), and penicillin/streptomycin (Gibco; Thermo Fisher Scientific, Inc., Waltham, MA, USA) to a suspension concentration of 10^6^ cells/mL. The suspension (200 μL) was then transferred onto coverslips attached to the wells of 35 mm plastic Petri dishes (MatTek IVLSL, Bratislava, Slovakia). The dishes were precoated with polyethyleneimine (1 mg/mL, 30 min, PEI not bound to the glass was washed with deionized sterile water 2 × 1 mL). One hour later, 1.5 mL of neurobasal medium containing 2% Supplement B27 and 0.5 mM L-glutamine was added. Cells were kept in an incubator at 37 °C, with 95% air + 5% CO_2_ and relative humidity of 100%. Cytosine beta D arabinofuranoside (Ara C, 5 μM; Sigma-Aldrich; Merck, St. Louis, MO, USA) was added to the medium for one day to prevent glial cell proliferation. Every three days, the cells were fed by replacing 1/3 of the old medium with the new medium. For cells phenotyping, cultures were stained with specific primary antibodies to β-tubulin (Invitrogen; Thermo Fisher Scientific, Waltham, MA, United States; Catalog # PA5-85639) and GFAP proteins (Thermo Fisher Scientific, Waltham, MA, United States; Catalog # OPA1-06100) as previously described [19]. Cultures with a percentage of neurons of more than 90% were used in experiments 10–12 days after plating (10–12 days in culture, DIV). Before each experiment, the bottoms of the cells and plates were washed ten times out of the B27 supplement with a buffer containing: (mM): 135 NaCl, 5 KCl, 2 CaCl_2_, 1 MgCl_2_, 20 HEPES, 5 d-glucose; pH 7.4. Cells were kept in this buffer for one hour before each experiment. Cells were kept in an incubator (37 °C, 95% air + 5% CO_2_, relative humidity 100%) in NBM. The composition of the NMB culture medium for bark cells (per 100 mL of the finished solution): 1 mL of gutamax (GlutaMax; Gibco; Thermo Fisher Scientific, Inc., Waltham, MA, USA), 2 mL of Supplement B-27, 1 mL of antibiotic/antimycotic (penicillin/streptomycin; Gibco; Thermo Fisher Scientific, Inc., Waltham, MA, USA), 96 mL of NMB solution. Cultures were used 10–12 days after planting (10–12 DIV).

### 4.2. Detection of Living, Apoptotic and Necrotic Cells Using Vital Fluorescent Dyes

In morphological studies of the viability cortical neurons in rats exposed to neurotoxic doses of glutamate, the following concentrations of fluorescent probes were used: Hoechst 33342—10 μg/mL; Syto-13—1 μM; ethidium homodimer (EthD-1)—3 μM. Staining was performed by adding stock solutions (all probes were diluted in dimethyl sulfoxide) to cells in saline buffer and incubated for 15 min at room temperature. The cultures were then rinsed with the same buffer and placed on the Evos FL Auto Life technologies fluorescent microscope stage. The Hoechst 33342 signal was recorded using a DAPI cube (excitation 360 nm, emission 447 nm). The Syto-13 signal was recorded using a GFP cube (excitation 470 nm, emission 525 nm). The EthD-1 signal was recorded using an RFP cube (530 nm excitation, 593 nm emission). Cells that had a blue-stained nucleus (intact or fragmented) and did not stain with Syto-13 (i.e., green) were interpreted as apoptotic. Cells that had a nucleus stained with EthD-1 (i.e., red) were interpreted as necrotic. Cells stained with Syto-13 (i.e., green) and had a nucleus stained with Hoechst 33342 were interpreted as living (cytoplasm stained with Syto-13 green and a weakly fluorescent nucleus by Hoechst). At least 1000 cells in randomly selected areas were counted. The average number of all cells in the control group was taken as 100%. The results obtained were analyzed using a two-way ANOVA test and the data were presented as the +/− standard error of the mean.

### 4.3. Measurement of [Ca^2+^]_i_ and Superoxide

To measure [Ca^2+^]_i,_ cortical neurons were loaded with a low-affinity Ca^2+^ indicator, Fura-FF, in the form of acetoxymethyl ester (in Fura-FF/AM-form, 2 μM, 60 min, 37 °C) in the presence of a non-ionic detergent, Pluronic F-127 (0.02%) which was acquired from Molecular Probes (Thermo Fisher Scientific, Waltham, MA, USA) to facilitate the penetration of Fura-FF into the cells. We used the low-affinity calcium dye Fura-FF in the experiments because long-term exposure to high doses of Glu (100 µM) results in such a high intracellular calcium concentration ([Ca^2+^]_i_) that high-affinity probes, such as Fura-2/AM, are rapidly saturated with calcium and does not reflect true changes in [Ca^2+^]_i_ [5,35]. Fura-FF has been repeatedly used to measure [Ca^2+^]_i_, including in experiments on Glu excitotoxicity [10,28,29]. Comparison of fluorescent images of neurons stained with Fura-FF and the Rh123 voltage-sensitive probe revealed a fundamental difference in the distribution of these dyes [28]. In our recent work [10], we described in detail the changes in [Ca^2+^]_i_ in different phases of OCD and presented corresponding images proving that Fura-FF AM penetrates well into the cell and is fairly evenly distributed in the cytosol. Mitochondria in cells (in neurons in particular) are able to accumulate Fura-FF only when using concentrations of Fura-FF/AM that are 2 orders of magnitude higher than those that are usually used to load cells. Therefore, mitochondria can only be loaded in a state isolated from cells.

Fura-FF was tested on neurons precisely because under the action of Glu, such high [Ca^2+^]_i_ occur in the cytosol, and Fura-2, which is widely used in research, is quickly saturated with calcium and does not reflect true changes in [Ca^2+^]_i_ in the cytosol [5,44,45]. For simultaneous measurements of [Ca^2+^]_i_ and [O_2_^–•^], cells were loaded with 1 μM of dihydroethidium (HE) fluorescent probe or triphenyl phosphonium linked hydroethidine (Mito-HE) fluorescent probe, a.k.a. MitoSOX Red, for the last 20 min of the “Fura-FF/AM loading period” in buffer at 37 °C. After loading, the cells were washed with saline of the following composition (in mM): NaCl—135; KCl—5; CaCl_2_—2; MgCl_2_—1; HEPES—20; glucose—5, pH 7.4. Insulin, at 100 nM, was added 5 min before 100 μM glutamate in Mg^2+^ free. To wash out Glu, a nominally Ca^2+^-free solution was used to exclude any pathways for Ca^2+^ entry from the buffer into the cytosol in the post-glutamate period. To assess the amount of Ca^2+^ accumulated by mitochondria, mitochondria in the final part of the experiments were depolarized using the FCCP protonophore (1 μM). To calibrate the maximum signal of the Ca^2+^ indicator, ionomycin (Iono, 2 µM) in the presence of 5 mM Ca^2+^ was added at the end of the experiment. The solutions were changed 3 times, 1 mL each. The cells were incubated on a microscope stage at 24–25 °C (Olympus IX-71, Tokyo, Japan). The Olympus IX-71 microscope was equipped with a 20x fluorite objective for measuring the fluorescence of various fluorescent probes. The light source is a Xenon arc lamp (175 W), the light of which alternately passes through interference filters installed in a computer-controlled wheel, which provides filter change in 50–200 ms (Lambda 10-2 Shutter, Instrument Co., Novato, CA, USA). A shutter between the lamp and the wheel with light filters block the radiation for the time when the signal is not recorded, preventing photo destruction of the object under study. The dichroic mirror reflects the exciting light toward the objective and transmits the radiation from the sample (colored cell) toward the detector. A lens focuses the excitation light on the sample and collects its fluorescence by directing it through a dichroic mirror to a detector. Cell fluorescence passed through the emission filter wheel, which was controlled synchronously with the excitation wheel. The image was detected using a CCD camera CoolSNAP HQ2 (Photometrics, Tucson, AZ, USA). The image analysis system was controlled with the MetaFluor 6.2 computer program (Molecular Devices, San Jose, CA, USA). The recording signals from cell indicators and images were recorded every 30 s. Fura-FF fluorescence was excited alternately at 340 and 380 nm and recorded at 525 nm. HE and Mito-HE excitation/emission fluorescence were 565/610 nm. Cell fluorescence was normalized relative to basal levels in resting cells; the typical interval between the acquisition of successive images (time-lapse) was 30 s.

### 4.4. Measurement of Oxygen Consumption Rates

Oxygen consumption rates (OCR) were measured by sequentially adding specific compounds from the Seahorse XF Cell Mito Stress Test Kit (Agilent Technologies, Inc., Santa Clara, CA, USA) using the Seahorse XFe24 Extracellular Flux Analyzer (Agilent Technologies, Inc., Santa Clara, CA, USA) according to the manufacturer’s protocol. Briefly, cells were incubated at a density of 50,000 cells/well in XFe24 microplates (Agilent Technologies, Inc., Santa Clara, CA, USA). Before the assay, the cells were washed thoroughly with cell medium (130 mM NaCl, 5 mM KCl, 2 mM CaCl_2_, 1 mM MgCl_2_, 20 mM HEPES, 5 mM Glucose, and 5 mM NaHCO_3_, at pH~7.4) and incubated in a CO_2_-free incubator at 37 °C for about 1 h. The microplates were then loaded into the XFe24 Analyzer. The microplate-based respirometry utilizes a 24-well plate format and quantifies the OCR at different times, following the addition of insulin, glutamate, or their vehicles as described earlier [28]. The non-mitochondrial oxygen consumption is the minimal OCR after 1 µM antimycin A or rotenone injection. Basal respiration is the difference between the last rate measurement before the first injection and the non-mitochondrial respiration rate. The maximal respiration is the difference between the maximum rate measurements of OCR after 1.5 µM carbonyl cyanide-4-(trifluoromethoxy)phenylhydrazone (FCCP) injection. The spare respiratory capacity is defined as the difference between maximal respiration and basal respiration. The baseline respiration, non-mitochondrial respiration, maximal respiration, and spare respiratory capacity were calculated via the Seahorse XF Cell Mito Stress Test Report Generator, which automatically calculates and reports assay parameters.

### 4.5. Statistical Analysis

Fluorescence measurement results processing was performed as previously described in detail [10]. All data are presented as the mean ± standard error of the mean (SEM) and were performed with the GraphPad Prism software. For comparing the difference between multiple groups, a one-way analysis of variance (ANOVA), followed by Tukey’s post-test for multiple comparisons was used. For comparing the difference in dynamics between groups, two-way ANOVA with repeated measures, followed by Tukey’s or Bonferroni’s post-test was used. Pearson’s correlation coefficient r was used to assess the correlations between groups. Statistically significant results are marked with asterisks, * *p* < 0.05; ** *p* < 0.01; *** *p* < 0.001; and **** *p* < 0.0001.

## 5. Conclusions

We found that insulin prevents glutamate-evoked intracellular and mitochondrial superoxides generation in primary cultures of rat cortical neurons by decreasing the glutamate-induced rise in [Ca^2+^]_i_. Given that superoxide causes neuron death, these results seem to explain the neuroprotective action of insulin in glutamate excitotoxicity. Insulin effects are also related to the improvement of spare respiratory capacity, the ability to produce additional ATP by oxidative phosphorylation in neurons during glutamate action.

## Data Availability

Not applicable.
